# Development of a personalized fall rate prediction model in community-dwelling older adults: a negative binomial regression modelling approach

**DOI:** 10.1186/s12877-023-03922-1

**Published:** 2023-03-30

**Authors:** Christina Wapp, Emmanuel Biver, Serge Ferrari, Philippe Zysset, Marcel Zwahlen

**Affiliations:** 1 ARTORG Center for Biomedical Engineering Research, University of Bern, Bern, Switzerland; 2 Division of Bone Diseases, Department of Medicine, Geneva University Hospitals and Faculty of Medicine, University of Geneva, Geneva, Switzerland; 3 Institute for Social and Preventive Medicine, University of Bern, Bern, Switzerland

**Keywords:** Falls, Fall rate, History of falls, Prediction, Count data

## Abstract

**Background:**

Around a third of adults aged 65 and older fall every year, resulting in unintentional injuries in 30% of the cases. Fractures are a frequent consequence of falls, primarily caused in individuals with decreased bone strength who are unable to cushion their falls. Accordingly, an individual’s number of experienced falls has a direct influence on fracture risk. The aim of this study was the development of a statistical model to predict future fall rates using personalized risk predictors.

**Methods:**

In the prospective cohort GERICO, several fall risk factor variables were collected in community-dwelling older adults at two time-points four years apart (T1 and T2). Participants were asked how many falls they experienced during 12 months prior to the examinations. Rate ratios for the number of reported falls at T2 were computed for age, sex, reported fall number at T1, physical performance tests, physical activity level, comorbidity and medication number with negative binomial regression models.

**Results:**

The analysis included 604 participants (male: 122, female: 482) with a median age of 67.90 years at T1. The mean number of falls per person was 1.04 and 0.70 at T1 and T2. The number of reported falls at T1 as a factor variable was the strongest risk factor with an unadjusted rate ratio [RR] of 2.60 for 3 falls (95% confidence interval [CI] 1.54 to 4.37), RR of 2.63 (95% CI 1.06 to 6.54) for 4 falls, and RR of 10.19 (95% CI 6.25 to 16.60) for 5 and more falls, when compared to 0 falls. The cross-validated prediction error was comparable for the global model including all candidate variables and the univariable model including prior fall numbers at T1 as the only predictor.

**Conclusion:**

In the GERICO cohort, the prior fall number as single predictor information for a personalized fall rate is as good as when including further available fall risk factors. Specifically, individuals who have experienced three and more falls are expected to fall multiple times again.

**Trial registration:**

ISRCTN11865958, 13/07/2016, retrospectively registered.

**Supplementary Information:**

The online version contains supplementary material available at 10.1186/s12877-023-03922-1.

## Background

Falls contribute substantially to increased morbidity and mortality in older people. Around 25—30% of persons aged 65 and older fall every year, and this number is increasing with advancing age [1, 2]. Approximately a third of all falls result in an injury [3], ranking falls as the leading cause of unintentional injuries and injury-related deaths [1, 4–7]. Thereby, fractures account for the most frequent consequence leading to disability [8]. While 5—15% of falls result in a fracture, the share of non-vertebral fractures caused by falls ranges between 59 – 96% and is site dependent [9]. Individuals who suffer from a fracture after falling are likely affected by decreased bone strength and are unable to cushion the fall [10]. Accordingly, the number of falls occurring to an individual influences the fracture risk [11]. In a meta-analysis including three cohorts, it was shown that the number of prior falls is improving fracture prediction over current used fracture risk assessment tools such as FRAX [12]. Accordingly, predicting not only the risk of falling but how many times an individual is expected to fall could improve fracture prediction in older adults.

Risk factors that have been associated with falling include increasing age, female gender, musculoskeletal deficits, gait and balance problems, a history of falls, fear of falling, vision impairment, cognitive deficits, urinary incontinence, medication, and comorbidities amongst many others [13–17]. To examine the risk of falling, various fall risk assessment tools and screening methods have been developed. A detailed overview of existing tools is provided in several reviews and meta-analyses [16, 18–24]. In summary, the assessments usually consist of performance tests and/or questionnaires that are designed to identify individuals at risk for falling. Thereby, when exceeding a defined threshold score value, individuals are considered as at risk of falling. So far, no single tool has been sufficient to successfully discriminate between fallers and non-fallers.

The association between fall risk factors and falling is often reported in the form of odds ratios derived with binary logistic regression. An alternative to measure the association are rate ratios. For this statistical method, not only the information whether a fall occurred or not is needed but the number of falls per individual is required. An expected fall rate can then be calculated. Count regression models belong to the family of generalized linear models and are used to estimate rates and rate ratios.

To our knowledge, only few studies have been conducted analysing the association between fall number and risk factors in terms of rate ratios [25–27]. Accordingly, the aim of this study was to develop a statistical model using a count regression approach for fall rate prediction and to investigate associations between the fall number and different fall risk factors for community-dwelling older adults.

## Methods

### Reporting guidelines

This publication followed the Transparent Reporting of a multivariable prediction model for individual Prognosis Or Diagnosis (TRIPOD) [28]. The completed checklist is available in the supplementary materials.

### Study participants

**Fig. 1:**
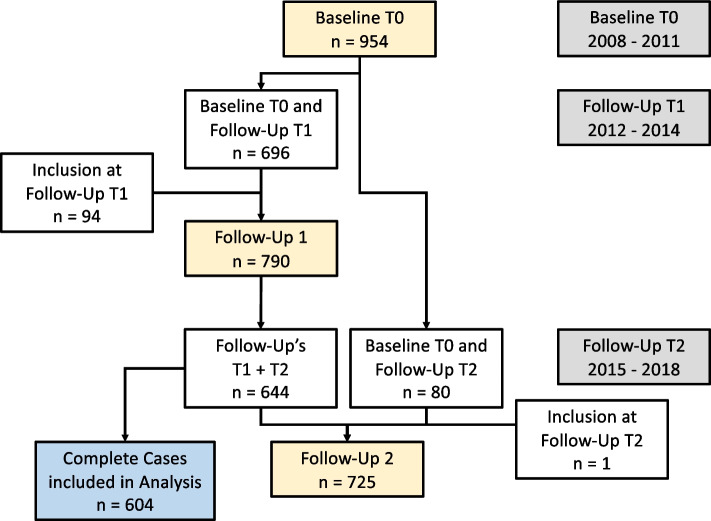
Flow of study participants in the GERICO study

The Geneva Retirees Cohort (GERICO) is a prospective cohort study conducted between 2008 and 2018 to identify risk factors for fall and fracture risk prediction in retired workers in the Geneva area (www.isrctn.com/ISRCTN11865958). From 2008 to 2011, healthy community-dwelling older adults were recruited using different strategies such as local newspaper advertisement, targeted mass mailing and advertisement at large local companies. After baseline examination (T0), participants were followed up the first time after 4 years (T1), and a second time after another 4 years (T2). Participants included in this study were of both sexes, aged between 63 and 67, around the time of their retirement, and living in the rural or urban Geneva area. Exclusion criteria were major comorbidities, in particular cancer treated within the last 5 years, chronic renal failure, liver or lung disease, corticosteroid therapy, primary hyperparathyroidism, Paget disease of bone, malabsorption or any neurological or musculoskeletal condition affecting bone health. The complete study design has been described previously [29, 30]. The present analysis included all participants that completed the two follow-up examinations at T1 and T2. The flow of participants in the study is presented in Fig. 1. The study protocol was approved by the University Hospitals Research Ethics Commission and written informed consent was provided by all participants.

### Sample size

Sample size calculation for the GERICO cohort was based on the primary outcome of the study, the number of incident fractures. No specific sample size calculation for the present analysis was conducted. All participants that completed the two follow-up visits at T1 and T2 were included in the current analysis (see Fig. 1).

### Variable selection

All covariates recorded in the GERICO cohort that have been associated with the risk of falling in literature and that are easily measurable (e.g., not requiring special equipment) and applicable in a clinical setting were included in the present analysis.

### Outcome

The number of falls was the outcome of interest. Participants were asked whether they had experienced any falls during the 12 months prior to the examination at T1 and T2 (also referred to as prior falls). The number of prior falls, the consequences (fracture, injurious fall requiring medical attention, injurious fall not requiring medical attention, uninjurious fall), and the activity during the fall (locomotion, transfer, run, sport, height/ladder, other) were recorded. A fall was defined as an event resulting in a person coming unintentionally to rest on the ground, floor, or any lower level. Extreme observations such as an individual falling five times and more, for example during sports or recreational activities, were not excluded since every single fall has the potential to result in an injurious outcome such as a fracture. The prior fall number reported at T2 was the independent variable.

### Predictors

#### History of falls

The number of experienced falls prior to T1 was included as predictor for the history of falls.

#### Demographic and anthropometric variables

Age, sex, standing height and body weight were recorded at follow-up examinations T1 and T2, and body mass index (BMI) was calculated accordingly.

#### Physical performance tests

Short physical performance battery (SPPB) is a test to assess lower extremity function in older adults [31]. Shortly, SPPB includes a balance test, the measurement of normal gait speed over 4 m distance, and the 5 times sit-to-stand test. Every subtest scores 4 points, reaching maximum 12 points. Hand grip strength (HGS) was measured with the JAMAR® Hand Dynamometer device. Participants were sitting on a chair with the elbow at a 90° flexion position. Three measurements were performed for each hand and the maximum value of the dominant or non-dominant hand was chosen. The one-legged stance test (OLST), also known as single-legged stance test, is a test assessing balance. Participants had to stand on one leg with crossed arms and eyes open. The test was stopped when reaching 45 s. If the maximum time was not reached, a second measurement was obtained. Both legs were tested, and the best performance of the dominant leg was used for analysis. No distinction between participants requiring one or two attempts to reach the maximum time was made. All physical performance tests were obtained both at T1 and T2.

#### Physical activity

Physical activity was evaluated both at T1 and T2 by a face-to-face questionnaire that uses an inventory of 45 activities to estimate the time spent on usual walking, cycling, stair climbing, organized sports, and recreational activity over the past 12 months. The collected data were converted to physical activity energy expenditure (kilocalories (kcal) per day) using validated formulas developed by Ainsworth et al. [32].

#### Comorbidities and medication

Age unadjusted Charlson’s comorbidity index (CCI) [33], the number of comorbidities and the number of medications were recorded at T2 only.

### Statistical analysis

#### Handling of predictors

Age and BMI were included from follow-up T1. Physical performance tests (SPPB, HGS and OLST) were also included from follow-up T1 to avoid retrodiction. Physical activity was included from follow-up visit T2 since it covers the same period of observation as the outcome variable. CCI, comorbidity and medication number were included from follow-up T2. The continuous variables (age, BMI, HGS, physical activity, comorbidity and medication number) were standardized to a mean of zero and a standard deviation of one, resulting in the rate ratio estimates corresponding to a standard deviation increase. HGS was standardised separately for sex due to a big difference in the corresponding mean. The number of prior falls at T1 was treated as a factor variable with levels 1, 2, 3, 4 and ≥ 5. SPPB, OLST and CCI were dichotomized because of their irregular and unbalanced distribution. SPPB score was dichotomized into the intervals [0, 9] and [10, 12]. The time score reached in the OLST was dichotomized into the three intervals [1, 20], [21, 40] and [41, 45]. CCI was dichotomized into the intervals [0, 1] and [2, 8].

#### Missing data

We conducted a complete case analysis but report the number of missing values for all variables as well as the number of participants with missing values.

#### Descriptive statistics

All variables were summarized with the median and the interquartile range (IQR). Additionally, the range and the mean number of prior falls at T1 and T2 were derived. For factor variables, the number of participants and the percentage per category respectively intervals were calculated.

#### Model fit

The Poisson regression is the best-known count regression model and assumes equidispersion of the count data (the mean equals the variance). However, most count data is overdispersed (the variance exceeding the mean). An alternative distribution that can model the overdispersion is the negative binomial distribution. It is described with an additional dispersion parameter that allows the variance to exceed the mean. Additionally, the negative binomial distribution is suitable to model recurrent events such as multiple falls per person by modelling the Poisson mean with a gamma distribution, accounting for population heterogeneity [34, 35]. In a study analysing fall count data from four cohorts, the negative binomial distribution performed best to model such data [35].

Accordingly, rate ratios (RR) were computed with negative binomial regression models using the log link and corresponding 95% Wald confidence intervals (CI) were calculated. Three different model types were fit: (1) 11 univariable models including every predictor separately, (2) a global model, including all available predictors described above, and (3) a subset model including age, sex, fall number reported at T1, SPPB, physical activity level and CCI. The covariates of the subset model were selected so that risk factors from different domains were represented while requiring as little time as possible when applied in a clinical setting. The number of falls reported at T2 was defined as the dependent variable. The generalized variance inflation factor (GVIF) was calculated for the global and subset model to detect the presence of multicollinearity among predictors [36]. GVIF is comparable to the variance inflation factor (VIF) when transformed by${( )}^{-1/2(n-p)}$. A transformed GVIF of ≤ 2.5 is acceptable [37]. Dispersion statistics was calculated with Pearson's Chi2 dispersion statistic given as $\frac{1}{(n-p)}{\sum }_{i=1}^{n}\frac{{e}_{i}}{Var(\widehat{{y}_{i}})}$ with *n* as the number of observations, *p* as the number of parameters included in the model [38]. A dispersion statistic greater than one indicates overdispersion, resulting in underestimation of standard errors of coefficient estimates and subsequently in too narrow confidence intervals [39].

To ensure that observations with high fall numbers do not bias the coefficient estimates, all models were re-fitted excluding observations with 5 or more falls either at T1 or T2.

#### Model comparison and prediction accuracy

Models were compared with the log-likelihood and the Bayesian information criteria (BIC). The error in a regression model is defined as ${{e}_{i}=y}_{i}-\widehat{{y}_{i}}$ with ${y}_{i}$ as the reported number of falls and $\widehat{{y}_{i}}$ as the models predicted number of falls for the $i$ th individual. Predictive performance was measured with the logarithmic score, the Brier score and the mean absolute error [40]. Internal model validation was conducted by calculating the mean absolute error for the test data with leave-one-out cross-validation. In count regression models with a small expected mean, residuals are usually not normally distributed [41]. Therefore, we also report the median and the interquartile range of the cross-validated residuals. Additionally, a marginal calibration diagram was derived to compare the actual number of individuals per fall number category at T2 to the predicted number of persons per fall number category based on the leave-one-out-prediction distribution in form of a hanging rootogram [40, 42].

#### Software

All statistical analysis was computed with R version 4.2.2.

## Results

Nine hundred fifty-four participants were included and participated in the baseline examination at T0. Thereof, 644 participants completed the two follow-up examinations at T1 and T2. 40 (6.2%) participants were excluded due to missing data, resulting in 604 observations included in the analysis (Fig. 1). Examination of excluded observations revealed no difference to the data used for analysis.

With a percentage of 79.8, most of the participants were female. Median (IQR) age was 67.90 (66.50, 69.03) years at T1, and median (IQR) BMI was 24.79 (22.26, 27.70) kg/m^2^. The median (IQR) SPPB score was 12.00 (12.00, 12.00) with 94.0% of observations reaching 10—12 points. Median (IQR) HGS was 28.60 (25.10, 33.70) kg. The difference in sex was 17.70 kg, resulting in a median (IQR) of 27.05 (24.33, 29.98) kg for female and 44.75 (40.20, 50.68) kg for male participants. Median (IQR) OLST time was 35.45 (17.22, 45.00) seconds. Median (IQR) kcal/day as a measure of physical activity was 291.29 (196.54, 434.27). The majority of CCI were observed in the interval 0.00 – 1.00 (94.9%). Median comorbidity and medication number were 2.00 with an IQR of (1.00, 3.00) and (1.00, 4.00), respectively. Table 1 presents the summary of all variables included in the analysis in detail.

**Table 1:** Summary of predictor variables and the outcome variable, the fall number at T2

Variable	Level and measure	Value	NA’s
Sex			0
	Female, n (%)	482 (79.8)	
	Male, n (%)	122 (20.2)	
*Assessed at T1*			
Fall number			4
	mean	1.04	
	median (IQR)	0.00 (0.00, 1.00)	
	min – max	0 – 20	
	0 falls, n (%)	310 (51.3)	
	1 falls, n (%)	174 (28.8)	
	2 falls, n (%)	57 (9.4)	
	3 falls, n (%)	31 (5.1)	
	4 falls, n (%)	9 (1.5)	
	≥ 5 falls, n (%)	23 (3.8)	
Age [years]			0
	median (IQR)	67.90 (66.50, 69.03)	
BMI [kg/m^2^]			1
	median (IQR)	24.79 (22.26, 27.70)	
SPPB (score 0–12)			16
	median (IQR)	12.00 (12.00, 12.00)	
	[0—9], n (%)	36 (6.0)	
	[10-12], n (%)	568 (94.0)	
HGS [kg]			16
	median (IQR)	28.60 (25.10, 33.70)	
OLST [s]			17
	median (IQR)	35.45 (17.22, 45.00)	
	[1-20], n (%)	177 (29.3)	
	[21-40], n (%)	149 (24.7)	
	[41-45], n (%)	278 (46.0)	
*Assessed at T2*			
Fall number			8
	mean	0.70	
	median (IQR)	0.00 (0.00, 1.00)	
	min – max	0—24	
	0 falls, n (%)	370 (61.3)	
	1 falls, n (%)	152 (25.2)	
	2 falls, n (%)	55 (9.1)	
	3 falls, n (%)	11 (1.8)	
	4 falls, n (%)	8 (1.3)	
	≥ 5 falls, n (%)	8 (1.3)	
Physical activity [kcal/day]			2
	median (IQR)	291.29 (196.54, 434.27)	
CCI (score)			1
	median (IQR)	0.00 (0.00, 0.00)	
	[0—1], n (%)	573 (94.9)	
	[2-8], n (%)	31 (5.1)	
Comorbidity (number)			0
	median (IQR)	2.00 (1.00, 3.00)	
Medication (number)			0
	median (IQR)	2.00 (1.00, 4.00)	

*Abbreviations*: *NA's* Missing data, *IQR* Interquartile range, *n* Number, % percentage, *BMI* Body mass index, *SPPB* Short physical performance battery, *HGS* Hand grip strength, *OLST* One-legged stance test, *CCI* Charlson's comorbidity index

**Fig. 2:**
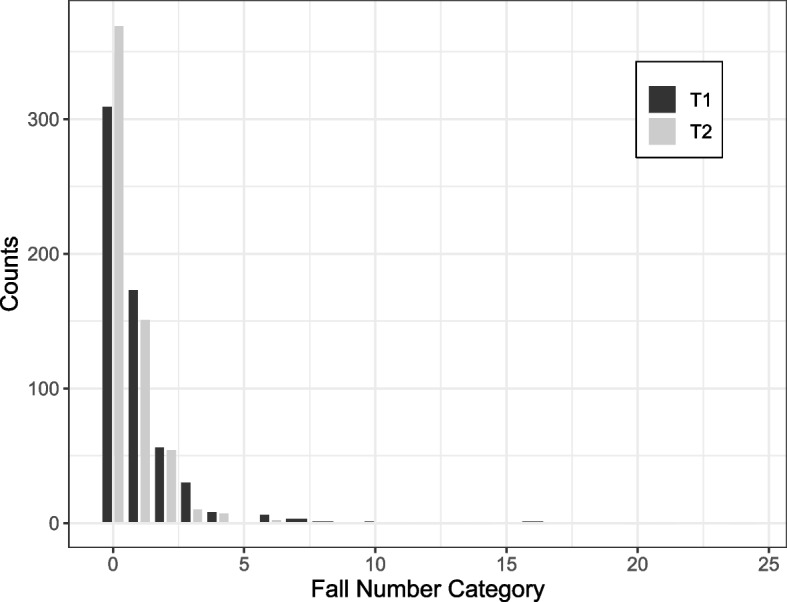
Distribution of the reported fall numbers at T1 and T2

The mean fall number at T1 was 1.04 and decreased to 0.70 falls at T2. At T1, 48.7% of participants reported to have fallen at least once in the previous 12 months, and 19.9% fell multiple times. This number decreased to 38.7% experiencing at least one fall and 13.6% falling multiple times at T2. Figure 2 presents the number of individuals per fall number category at T1 and T2. The reported numbers ranged from 0 to 20 for T1 and from 0 to 24 for T2. 220 participants reported no falls at T1 and T2, and 144 reported falls at T1 and T2. 150 persons fell before T1 but not before T2, and 90 participants experienced no falls prior to T1 but reported falls at T2. The total reported fall number was 630 at T1 and 425 at T2. At T1 and T2, in 9% of the cases, the falls resulted in injuries that required medical attention. 34% at T1 and 36% at T2 caused injuries requiring no medical attention and 54% at T1 and 50% at T2 had no consequences. 2% of the falls caused a fracture at T1. This number increased to 4% at T2. 53% of the falls reported at T1 and 50% at T2 occurred during locomotion or transfer, while 36% at T1 and 44% at T2 happened during running or sports activities. Falls from heights or ladders accounted for 1% at T1 and 4% at T2 of the falls. The rest of the cases was unclear or not reported.

RR estimates and 95% CI of the predictors for the univariable models, the global and the subset model are presented in Table 2. Reference levels of factor variables are reported with a RR = 1.00.

**Table 2:** Rate ratios and 95% confidence interval for the univariable, the global and the subset models

Variables	Univariable	Global	Subset
Sex			
Male	1.00	1.00	1.00
Female	0.67 (0.48, 0.94)	0.94 (0.67, 1.32)	0.94 (0.67, 1.31)
*Assessed at T1*			
Age [years]]	1.07 (0.92, 1.23)	1.08 (0.94, 1.23)	1.07 (0.94, 1.22)
Fall number			
0	1.00	1.00	1.00
1	1.67 (1.23, 2.27)	1.65 (1.22, 2.23)	1.64 (1.21, 2.22)
2	1.16 (0.71, 1.90)	1.20 (0.74, 1.95)	1.20 (0.74, 1.96)
3	2.60 (1.54, 4.37)	2.67 (1.61, 4.45)	2.56 (1.53, 4.31)
4	2.63 (1.06, 6.54)	3.31 (1.38, 7.98)	2.69 (1.08, 6.65)
≥ 5	10.19 (6.25, 16.60)	7.70 (4.74, 12.51)	8.92 (5.47, 14.57)
BMI [kg/m^2^]	0.95 (0.82, 1.10)	0.96 (0.83, 1.10)	-
SPPB (score 0 – 12)			
[10–12]	1.00	1.00	1.00
[0—10]	0.82 (0.44, 1.53)	0.88 (0.49, 1.57)	0.94 (0.53, 1.65)
HGS [kg]	1.16 (1.01, 1.33)	1.08 (0.95, 1.22)	-
OLST [s]			
[41–45]	1.00	1.00	-
[1–20]	1.11 (0.81, 1.54)	1.17 (0.85, 1.62)	-
[21–40]	0.59 (0.40, 0.86)	0.71 (0.49, 1.01)	-
*Assessed at T2*			
Physical activity [kcal/day]	1.29 (1.13, 1.47)	1.11 (0.97, 1.26)	1.12 (0.98, 1.28)
CCI (score)			
[0—1]	1.00	1.00	1.00
[2–8]	2.08 (1.18, 3.67)	1.40 (0.81, 2.43)	1.38 (0.81, 2.36)
Comorbidity (number)	0.99 (0.86, 1.14)	1.18 (0.99, 1.41)	-
Medication (number)	0.82 (0.71, 0.95)	0.82 (0.68, 0.98)	-

Reference levels of factor variables are indicated with a rate ratio = 1.00. For continuous variables, rate ratios correspond to a standard deviation increase

*Abbreviations*: *BMI* Body mass index, *SPPB* Short physical performance battery, *HGS* Hand grip strength, *OLST* One-legged stance test, *CCI* Charlson’s comorbidity index

For the univariable models, the fall number at T2 was associated with female sex (RR 0.67, 95% CI: 0.48 to 0.94, male sex as reference category); with the prior fall number reported at T1 (e.g., ≥ 5 falls: RR 10.19, 95% CI: 6.25 to 16.60, 0 falls as reference category); with the HGS (RR 1.16, 95% CI: 1.01 to 1.33); with the OLST when reaching 21 – 40 s (RR 0.59, 95% CI: 0.40 to 0.86, 41 – 45 s as reference category); with the physical activity level (RR 1.29, 95% CI: 1.13 to 1.47); with the CCI when scoring 2 – 8 (RR 2.08, 95% CI: 1.18 to 3.67, a score of 0 – 1 as reference category); and with the number of medication (RR 0.82, 95% CI: 0.71 to 0.95). In the global model, associations were found for the prior fall number at T1 (e.g., 3 falls: RR 2.67, 95% CI: 1.61 to 4.45, 0 falls as reference category); and the number of medication (RR 0.82, 95% CI: 0.68 to 0.98). For the subset model, the number of falls at T2 was associated with the prior fall number at T1 (e.g., 4 falls: RR 2.69, 95% CI: 1.08 to 6.65, 0 falls as reference category). All other predictors were not associated with the fall number reported at T2.

**Fig. 3:**
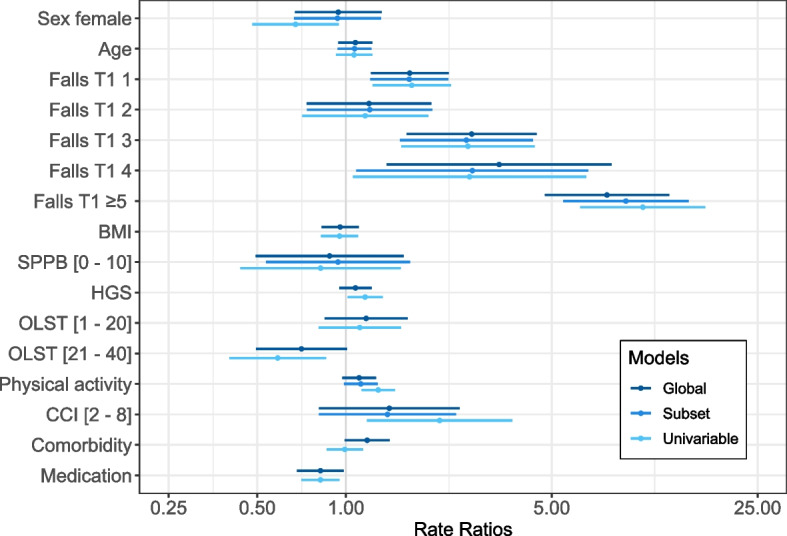
Rate ratios presented in a forest plot for the univariable, the global and the subset models. Confidence bands correspond to 95% confidence intervals. For continuous variables, rate ratios correspond to a standard deviation increase. Reference levels for factor variables: male sex; Falls T1 0; SPPB [11, 12]; OLST [41–45]; CCI [0 – 1]. Abbreviations: BMI = body mass index, SPPB = short physical performance battery, HGS = hand grip strength, OLST = one-legged stance test, CCI = Charlson’s comorbidity index

Figure 3 depicts the rate ratios and their 95% CI presented in Table 2, visualizing the differences in the estimates between the models.

The baseline rates represent the expected fall number for an individual belonging to the reference category of factor variables (e.g., sex: male), or belonging to the mean value of a continuous variable. For the univariable models, the baseline rate ranged from 0.67 to 0.95 (supplementary material, Table S1). The baseline rate for the global model was 0.44 (95% CI 0.31 to 0.64), for the subset model 0.44 (95% CI 0.31 to 0.62), and for the univariable model including the number of falls at T1 0.42 (95% CI 0.35 to 0.52).

The coefficient estimates of the models excluding all observations with ≥ 5 falls at T1 or T2 were comparable to the here presented results (supplementary material, Table S2).

The following results are only presented for the univariable model including fall number at T1 as factor variable (referred to as falls model), the global model, and the subset model. The complete list of all univariable models can be found in the supplementary material (supplementary material, Table S1).

Measures for model fit and model performance of the global, subset and the fall model are shown in Table 3. The log-likelihood was best for the global model followed by the subset model and lowest for the falls model (global: -630.79, subset: -637.59, falls: -640.75). Considering the BIC, the falls model performed best (global: 1376.85, subset: 1352.03, falls: 1326.33). The logarithmic score was best for the global model, and similar for the subset and falls model (global: 1.04; subset: 1.06; falls: 1.07), and the Brier score was also best for the global model (global: -0.47; subset: -0.46; falls: -0.45). The mean absolute error was lowest for the global model, followed by the subset and the falls model (global: 0.79; subset: 0.81; falls: 0.82). The cross-validated mean absolute error was for all models slightly higher compared to the apparent error (global: 0.83; subset: 0.84; falls: 0.84). The median absolute error was lower compared to the mean absolute error, showing that the residuals are not normally distributed and skewed towards zero. It was again comparable for all three models (global: 0.51; subset: 0.53; falls: 0.50). All here presented measures for the other univariable models can be found in Table S1 in the supplementary material. In summary, none of the other models performed as good as the global, the subset or the falls model.

**Table 3:** Model comparison and prediction measures for the global, the subset and the univariable falls model

Model	LL	BIC	LS	BS	MAE	CV MAE	CV median absolute error (IQR)
Global	-630.79	1376.85	1.04	-0.47	0.79	0.83	0.51 (0.38, 0.72)
Subset	-637.59	1352.03	1.06	-0.46	0.81	0.84	0.53 (0.39, 0.69)
Falls	-640.75	1326.33	1.07	-0.45	0.82	0.84	0.50 (0.42, 0.71)

*Abbreviations*: *LL* Log-likelihood, *BIC* Bayesian information criteria, *LS* Logarithmic score, *BS* Brier score, *MAE* Mean absolute error, *CV* Cross-validated, *IQR* Interquartile range

**Fig. 4:**
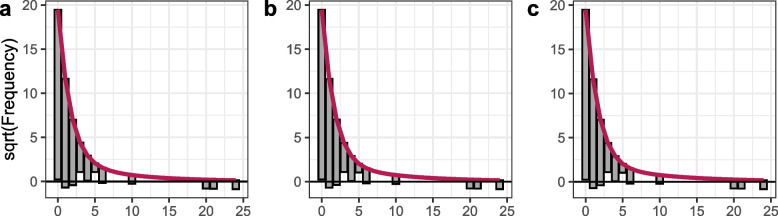
Hanging rootograms for (**a**) the global, (**b**) the subset and (**c**) the univariable fall model. Hanging rootograms as marginal calibration plots show the deviations between the actual (grey bars) and predicted (red line) number of individuals per fall number category

Figure 4 presents the models’ marginal calibration plots, comparing the number of participants per reported fall number category at T2 (represented by the grey bars) to the predicted number of individuals per fall number category (red line) of (a) the global, (b) the subset and (c) the falls model based on the cross-validated leave-one-out prediction distribution as hanging rootograms. Deviations between actual and predicted numbers become visible when focusing on the position of the bar’s lower ends: bars reaching the negative frequency range indicate underestimation of the predicted counts, while bars not reaching the x-axis represent overestimated frequencies. The predicted size of individuals per fall number category were similar for all three models. The models underestimated the number of individuals who experienced two and three fall events, while the number of individuals with zero falls and higher fall numbers were overestimated. Extreme events (e.g., an individual falling 20 times) could not be accurately predicted.

Pearson’s Chi2 dispersion statistic was comparable for the three models (global: 1.05, subset: 1.05, falls: 1.08). ${GVIF}^{-1/2(n-p)}$ was smaller than 2.5 for all covariates in the global and subset model, indicating no multicollinearity.

To understand how the models are used for predicting an individual’s fall rate, this is demonstrated for the subset model. The rate ratios presented in Table 2 are reported on the response scale. To make predictions, it is easiest to transform the rate ratios to the link scale by taking the log. Following form that, the subset model writes as

$$\log\left(fall\;rate\right)=\log\left(0.44\right)+log\left(0.94\right){\bullet I}_{Sex=female}+log\left(1.07\right)\bullet{Age}_{standardised}+log\left(1.64\right)\bullet I_{Falls=1}+log\left(1.20\right)\bullet I_{Falls=2}+\left(2.56\right)\bullet I_{Falls=3}+log\left(2.69\right)\bullet I_{Falls=4}\ast log\left(8.92\right)\bullet I_{Falls\geq5}+log\left(0.94\right)\bullet I_{SPPB=\left[0-10\right]}+\log(1.12)\bullet{Activity}_{standardised}+log(1.38)\bullet I_{CCI=\lbrack2-8\rbrack}$$

where $I$ is the indicator function given as

$$I_{condition}=\left\{\begin{array}{cc}1,&condition\;is\;true\\0,&otherwise\end{array}\right.$$

As an example, the fall rate of a female individual with a standardized age of 1.5, having experienced 3 falls within the last 12 months, reaching an SPPB score of 11, with a standardized physical activity level of -1.5 and a CCI of 3 calculates as

$$\log\left(fall\;rate\right)=\log\left(0.44\right)+\log\left(0.94\right)\bullet1+log\left(1.07\right)\bullet1.5+\log\left(1.64\right)\bullet0+\log\left(1.20\right)\bullet0+\log\left(2.56\right)\bullet1+\log\left(2.69\right)\bullet0+\log\left(8.92\right)\bullet0+\log\left(0.94\right)\bullet0+\log\left(1.12\right)\bullet(-1.5)+\log\left(1.38\right)\bullet1$$

simplifying to

$$fall\;rate=0.44\ast0.94\ast1.11\ast2.56\ast0.84\ast1.38=1.36$$

## Discussion

This analysis examined the association of different fall risk factors and the number of falls reported within 12 months, and aimed to develop a model for personalized fall rate prediction. In contrast to most other cohort analyses, we derived rate ratios using negative binomial regression models.

The mean number of falls per person in the GERICO cohort was higher compared to other studies. For example, the federal office of Switzerland reported that 1 out of 4 adults aged 65 and older experienced a fall [2], while a questionary in the US resulted in 15.9% of people experiencing a fall [3]. However, the GERICO cohort is a comparably young and fit cohort, and many falls occurred during sports and recreative activities.

The main finding of our analysis is the strong association between the history of falls and future falls. Thereby, the more falls an individual experienced, the stronger the association was. This result is consistent with literature, reporting recurrent fallers to have a higher odds-ratio to fall again when compared to one-time fallers [17]. Interestingly, the rate ratio for falling when having experienced two previous falls is lower compared to one or three previous falls. While falling once might happen by chance, individuals who fell twice possibly become attentive to the risk of falling and initiate preventive measures. However, after having experienced three or multiple falls, the ability to prevent future fall events might be insufficient.

The association of other predictors in the univariable models with the number of falls was only partly congruent with other studies, reviews and meta-analyses assessing the risk of falling in community-dwelling older adults. We want to point out that the direct comparison with other cohort analyses must be handled with care, since differences in study design, participant's characteristics, assessment of tests and analysis methods need to be considered.

Compared to men, female participants were expected to fall less, while in literature the opposite is reported [17]. No association was found between age and the fall number in the GERICO cohort, although increasing age is a widely recognized risk factor for falls [1, 17]. Actually, the total number of reported falls in the previous 12 months as well as the percentage of fallers decreased from T1 to T2 in spite of 4 years advancing age. With its rather young median age and narrow age range, the GERICO cohort might not be suitable to detect such a relationship. A longer follow-up time and a wider age range might be required. In addition, we assume that the decrease in fall number and percentage from T1 to T2 reflects a decrease in recreational and sports activity of the study participants, resulting in fewer falls. Physical performance measures were also not associated to falls as reported in literature. The SPPB was not predictive for falls. However, this is not surprising when considering that more than 90% of the participants in the GERICO cohort scored 10—12 points. In another study examining the predictive value of the SPPB, it was reported that scores of less than 6 are associated with an increased fall risk in older adults [43]. Contrary to literature, HGS was positively associated with the number of falls [27, 44, 45]. Again, this might reflect the cohort’s fitness level, resulting in recreational and sport-related falls. Additionally, a study comparing the performance of HGS with hip muscle strength to discriminate between fallers and non-fallers reported HGS to be less accurate compared to assessments including lower limb strength [46]. The results from the OLST were not conclusive and related the opposite way than reported in literature [47]. As reported in a meta-analysis assessing balance tests for fall risk prediction, the test might not be sensitive enough to discriminate between fallers and non-fallers [47]. The positive association between physical activity and falls might be best explained by the fact that many falls in this study were induced during recreational and sports activities. Considering comorbidities and medication, further inconclusive associations were found. In contrast to the results of other studies, no association was found between the number of comorbidities and falls [27]. However, CCI as a measure of comorbidity was associated with the fall number in the GERICO cohort. Contrary to our expectations and other reported results, the number of medications was negatively associated with falls [27]. It is known that some medications are associated with an increase in fall risk [48], while others such as Vitamin D can have a preventive effect [49]. To better understand this finding, a detailed analysis of medication type would be required.

Considering the prediction accuracy of the models, particularly interesting is that the internally validated errors for the univariable model with the prior number of falls as the only predictor and the two multivariable models including additional predictors are comparable. Hence, the information included in the other predictors does not improve the model’s predictive performance. The reported error for the falls model is lower than in a recently published study presenting a prediction model for falls in community-dwelling older adults using a comparable analysis method (bootstrapped mean absolute error 0.88) [27]. In order to evaluate the model’s performance in detail, sensitivity analysis and external validation are required.

Although an error of less than one fall seems small, this variation could be critical to whether fall-prone individuals are correctly identified, especially in the lower range of fall numbers. With the ulterior motive to integrate a fall rate estimate in a fracture risk model, such a prediction error might have a considerable impact on subsequent fracture risk assessment.

The consequences of predicting a higher fall rate than effectively occurring seem less problematic compared to underestimating the number of expected falls. All three models presented here underestimated the number of individuals experiencing 1 and 2 falls while overestimating the number of 0 falls, bearing the risk of missing the identification of individuals who require fall prevention measures. Further predictive risk factors need to be identified that are sensitive enough to minimize such errors and improve prediction accuracy. Ideally, these predictors should also be suitable to identify first-time fallers without a history of falls.

In most fall risk assessments, not the number of previously experienced falls is recorded, but whether any falls have occurred at all [17, 19]. Based on our results, we believe that additional information on the number of prior falls has great potential to improve the identification of individuals at risk for falling at a manageable cost. Therefore, we encourage other researchers to additionally record the number of previously experienced falls over a clearly defined time-period.

### Limitations

This study and analysis has several limitations. First, the study design is not optimal for the development of a fall prediction model. A time interval of 4 years between the follow-up examinations at T1 and T2 is very long when physical performance parameters such as balance or muscle strength are examined. This makes it difficult to detect associations between fall risk predictors and the number of falls. Ideally, risk factors would be assessed at a baseline examination followed by an observation period in which the number of falls is recorded. Nevertheless, we chose to use the obtained data of the physical performance tests from T1 instead of T2 because we wanted to exclude the possibility of retrodiction (e.g., a participant performing medium in SPPB at T2 because of a recently experienced fall shortly before the examination). However, since comorbidity and medication were only assessed at T2, a certain risk of retrodiction for those variables could not be circumvented. Similarly, physical activity was assessed over the same time-period as the outcome variable, possibly resulting in a decreased activity level for individuals with severe falls at the beginning of this observation period. This again increases the likelihood of reverse causation.

Second, the participants were asked whether they had experienced any prior falls at the follow-up visits at T1 and T2 without knowing that this question will be asked. It was shown that self-reported retrospective recording of fall numbers might be inaccurate [50, 51]. Thirdly, not all domains of fall risk factors are covered with the available predictors. For example, no questions and tests considering fear of falling, vision or cognition have been included in the study protocol. Last, the GERICO cohort is a comparable young and fit cohort and is possibly not the best representation of older adults at risk of falling. Therefore, the study findings bear a potential lack of generalisability.

## Conclusion

In the GERICO cohort, the prior fall number as single predictor information for a personalized fall rate is as good as a model including all available fall risk factors. Specifically, individuals who have experienced three and more falls are expected to experience multiple falls again. Because falling is a complex phenomenon and a broad range of conditions influence whether or not a fall occurs, it seems reasonable that the complex circumstances under which a fall occurs are best reflected by the history of falls itself.

## Supplementary Information

**Additional file 1:** **Table S1.** Baseline rate ratio, dispersion statistics, model comparison and predictive performance measures for the univariable models. **Table S2.** Rate ratios and the corresponding 95 % confidence interval for the data set excluding extreme fall events (≥ 5 falls). TRIPOD Checklist: Prediction Model Development.

## Data Availability

The datasets used and/or analysed during the current study are available from the corresponding author upon reasonable request.

## Abbreviations

BIC: Bayesian information criteria
BMI: Body mass index
CCI: Charlson’s comorbidity index
CI: Confidence interval
GERICO: Geneva Retirees Cohort
GVIF: Generalized variance inflation factor
HGS: Hand grip strength
IQR: Interquartile range
OLST: One-legged stance test
RR: Rate ratio
SPPB: Short physical performance battery
VIF: Variance inflation factor
