# Supplementary material for: Development of a personalized fall rate prediction model in community-dwelling older adults: a negative binomial regression modelling approach
Source: BMC Geriatr. 2023 Mar 30;23:200. doi: 10.1186/s12877-023-03922-1 (PMC10064572; doi:10.1186/s12877-023-03922-1)
Supplement: Supplementary file 1 — Additional file 1: Table S1. Baseline rate ratio, dispersion statistics, model comparison and predictive performance measures for the univariable models. Table S2. Rate ratios and the corresponding 95 % confidence interval for the data set excluding extreme fall events (≥ 5 falls). TRIPOD Checklist: Prediction Model Development. [file 12877_2023_3922_MOESM1_ESM.docx]

**Supplementary materials**

*Tables*

Table S1. Baseline rate ratio, dispersion statistics, model comparison and predictive performance measures for the univariable models.

| Univariable Models | Baseline RR (CI) | PChi2D | LL | BIC | LS | BS | MAE | CV MAE | CV median absolute error (IQR) |
| --- | --- | --- | --- | --- | --- | --- | --- | --- | --- |
| Sex | 0.95 (0.71, 1.28) | 1.81 | -686.77 | 1392.75 | 1.14 | -0.44 | 0.86 | 0.87 | 0.64 (0.36, 0.96) |
| Age | 0.70 (0.61, 0.81) | 2.06 | -689.09 | 1397.38 | 1.14 | -0.44 | 0.86 | 0.86 | 0.69 (0.38, 0.75) |
| BMI | 0.70 (0.61, 0.81) | 2.03 | -689.24 | 1397.69 | 1.14 | -0.44 | 0.86 | 0.86 | 0.70 (0.41, 0.74) |
| SPPB | 0.71 (0.61, 0.82) | 2.04 | -689.23 | 1397.67 | 1.14 | -0.44 | 0.86 | 0.86 | 0.71(0.43, 0.71) |
| HGS | 0.70 (0.60, 0.80) | 1.87 | -687.66 | 1394.54 | 1.14 | -0.44 | 0.86 | 0.87 | 0.67 (0.48, 0.78) |
| OLST | 0.76 (0.62, 0.93) | 1.87 | -684.15 | 1393.91 | 1.13 | -0.45 | 0.85 | 0.85 | 0.76 (0.45, 0.85) |
| Activity | 0.68 (0.59, 0.79) | 1.86 | -683.46 | 1386.13 | 1.13 | -0.44 | 0.87 | 0.88 | 0.61 (0.49, 0.80) |
| CCI | 0.67 (0.58, 0.77) | 1.81 | -686.02 | 1391.25 | 1.14 | -0.44 | 0.87 | 0.87 | 0.67 (0.40, 0.67) |
| Comorbidity | 0.70 (0.61, 0.81) | 2.04 | -689.42 | 1398.04 | 1.14 | -0.44 | 0.86 | 0.87 | 0.70 (0.32, 0.71) |
| Medication | 0.69 (0.60, 0.80) | 1.76 | -685.93 | 1391.07 | 1.14 | -0.44 | 0.86 | 0.86 | 0.70 (0.46, 0.81) |

Abbreviations: RR = rate ratio, CI = 95 % confidence interval, PChi2D = Pearson’s Chi2 dispersion statistic, LL = log-likelihood, BIC = Bayesian information criteria, LS = logarithmic score, BS = Brier score, MAE = mean absolute error, CV = cross-validated, IQR = interquartile range.

Table S2. Rate ratios and the corresponding 95 % confidence interval for the data set excluding extreme fall events (≥ 5 falls).

| Variables | | | | Univariable | Global | Subset |
| --- | --- | --- | --- | --- | --- | --- |
| Sex | | | |  |  |  |
|  | male | | | 1.00 | 1.00 | 1.00 |
|  | female | | | 0.84 (0.62, 1.14) | 0.94 (0.68, 1.31) | 0.91 (0.66, 1.26) |
| *Assessed at T1* | | | | | | |
| Age [years] | | | | 1.02 (0.90, 1.16) | 1.02 (0.89, 1.15) | 1.02 (0.90, 1.15) |
| Fall number | | | |  |  |  |
|  | 0 | | | 1.00 | 1.00 | 1.00 |
|  | 1 | | | 1.53 (1.15, 2.02) | 1.52 (1.15, 2.01) | 1.50 (1.13, 1.99) |
|  | 2 | | | 1.20 (0.77, 1.88) | 1.20 (0.77, 1.87) | 1.22 (0.78, 1.90) |
|  | 3 | | | 2.86 (1.12, 3.09) | 1.86 (1.12, 3.07) | 1.86 (1.12, 3.09) |
|  | 4 | | | 2.72 (1.28, 5.80) | 3.03 (1.43, 6.38) | 2.75 (1.29, 5.84) |
| BMI [kg/m^2^] | | | | 0.98 (0.86, 1.11) | 0.96 (0.83, 1.10) | - |
| SPPB (score 0 – 12) | | | |  |  |  |
|  | | [10 - 12] | | 1.00 | 1.00 | 1.00 |
|  | | [0 - 10] | | 0.83 (0.47, 1.47) | 0.77 (0.43, 1.36) | 0.83 (0.47, 1.46) |
| HGS [kg] | | | | 1.01 (0.98, 1.15) | 1.01 (0.89, 1.14) | - |
| OLST [s] | | | |  |  |  |
|  | | [41 - 45] | | 1.00 | 1.00 | - |
|  | | [1 - 20] | | 1.05 (0.78, 1.40) | 1.05 (0.76, 1.45) | - |
|  | | [21 - 40] | | 0.79 (0.57, 1.09) | 0.78 (0.56, 1.09) | - |
| *Assessed at T2* | | | | | | |
| Physical activity [kcal/day] | | | | 1.08 (0.95, 1.11) | 1.06 (0.92, 1.21) | 1.05 (0.92, 1.21) |
| CCI (score) | | | |  |  |  |
|  | | | [0 - 1] | 1.00 | 1.00 | 1.00 |
|  | | | [2 - 8] | 1.31 (0.77, 2.21) | 1.27 (0.74, 2.18) | 1.24 (0.73, 2.10) |
| Comorbidity (number) | | | | 1.09 (0.96, 1.24) | 1.23 (1.03, 1.46 | - |
| Medication (number) | | | | 0.98 (0.86, 1.11) | 0.84 (0.70, 1.00) | - |

Reference levels of factor variables are indicated with a rate ratio = 1.00. For continuous variables, rate ratios correspond to a standard deviation increase. Abbreviations: BMI = body mass index, SPPB = short physical performance battery, HGS = hand grip strength, OLST = one-legged stance test, CCI = Charlson’s comorbidity index.

TRIPOD Checklist: Prediction Model Development
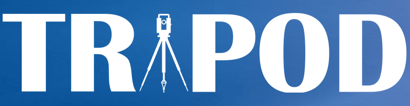


| **Section/Topic** | **Item** | **Checklist Item** | **Page** |
| --- | --- | --- | --- |
| **Title and abstract** | | | |
| Title | 1 | Identify the study as developing and/or validating a multivariable prediction model, the target population, and the outcome to be predicted. | 1 |
| Abstract | 2 | Provide a summary of objectives, study design, setting, participants, sample size, predictors, outcome, statistical analysis, results, and conclusions. | 1 |
| **Introduction** | | | |
| Background and objectives | 3a | Explain the medical context (including whether diagnostic or prognostic) and rationale for developing or validating the multivariable prediction model, including references to existing models. | 2 |
|  | 3b | Specify the objectives, including whether the study describes the development or validation of the model or both. | 2 |
| **Methods** | | | |
| Source of data | 4a | Describe the study design or source of data (e.g., randomized trial, cohort, or registry data), separately for the development and validation data sets, if applicable. | 2 |
|  | 4b | Specify the key study dates, including start of accrual; end of accrual; and, if applicable, end of follow-up. | 2 |
| Participants | 5a | Specify key elements of the study setting (e.g., primary care, secondary care, general population) including number and location of centres. | 2 |
|  | 5b | Describe eligibility criteria for participants. | 2 |
|  | 5c | Give details of treatments received, if relevant. | - |
| Outcome | 6a | Clearly define the outcome that is predicted by the prediction model, including how and when assessed. | 3 |
|  | 6b | Report any actions to blind assessment of the outcome to be predicted. | - |
| Predictors | 7a | Clearly define all predictors used in developing or validating the multivariable prediction model, including how and when they were measured. | 3, 4 |
|  | 7b | Report any actions to blind assessment of predictors for the outcome and other predictors. | - |
| Sample size | 8 | Explain how the study size was arrived at. | 2 |
| Missing data | 9 | Describe how missing data were handled (e.g., complete-case analysis, single imputation, multiple imputation) with details of any imputation method. | 4 |
| Statistical analysis methods | 10a | Describe how predictors were handled in the analyses. | 4 |
|  | 10b | Specify type of model, all model-building procedures (including any predictor selection), and method for internal validation. | 4 |
|  | 10d | Specify all measures used to assess model performance and, if relevant, to compare multiple models. | 4, 5 |
| Risk groups | 11 | Provide details on how risk groups were created, if done. | - |
| **Results** | | | |
| Participants | 13a | Describe the flow of participants through the study, including the number of participants with and without the outcome and, if applicable, a summary of the follow-up time. A diagram may be helpful. | 3, 5 |
|  | 13b | Describe the characteristics of the participants (basic demographics, clinical features, available predictors), including the number of participants with missing data for predictors and outcome. | 5, 6 |
| Model development | 14a | Specify the number of participants and outcome events in each analysis. | 5 |
|  | 14b | If done, report the unadjusted association between each candidate predictor and outcome. | 7 |
| Model specification | 15a | Present the full prediction model to allow predictions for individuals (i.e., all regression coefficients, and model intercept or baseline survival at a given time point). | 7 |
|  | 15b | Explain how to the use the prediction model. | 9 |
| Model performance | 16 | Report performance measures (with CIs) for the prediction model. | 8 |
| **Discussion** | | | |
| Limitations | 18 | Discuss any limitations of the study (such as nonrepresentative sample, few events per predictor, missing data). | 10 |
| Interpretation | 19b | Give an overall interpretation of the results, considering objectives, limitations, and results from similar studies, and other relevant evidence. | 9, 10 |
| Implications | 20 | Discuss the potential clinical use of the model and implications for future research. | 10 |
| **Other information** | | | |
| Supplementary information | 21 | Provide information about the availability of supplementary resources, such as study protocol, Web calculator, and data sets. | 11 |
| Funding | 22 | Give the source of funding and the role of the funders for the present study. | 11 |
